# Concrete Damaged Plasticity-Based Analysis of Damage and Stiffness Degradation in Cooling Tower Shells Under Spatially Variable Seismic Loading

**DOI:** 10.3390/ma19102139

**Published:** 2026-05-20

**Authors:** Paweł Boroń, Joanna Maria Dulińska

**Affiliations:** Faculty of Civil Engineering, Cracow University of Technology, 31-155 Krakow, Poland; joanna.dulinska@pk.edu.pl

**Keywords:** material-driven seismic analysis, concrete damage plasticity model, nonlinearity of concrete, damage evolution, cooling tower, spatially varying seismic motion, SVEGM

## Abstract

This study investigates the seismic response of a natural draft reinforced concrete cooling tower subjected to spatially varying earthquake ground motion, with particular emphasis on nonlinear material behavior, damage evolution, and stiffness degradation. The analysis is based on a constitutive description of concrete using the Concrete Damaged Plasticity (CDP) model, enabling the representation of tensile cracking, compressive crushing, and irreversible plastic deformation under cyclic dynamic loading. Two structural configurations of the lower shell region–a locally thickened shell and a bottom ring-stiffened solution–are examined from the perspective of material performance and damage control. Spatially varying seismic excitation is defined using a real earthquake record from the Carpathian Flysch region, with wave passage and incoherence effects calibrated from in-situ measurements. Nonlinear time-history analyses, performed to capture the coupling between material degradation mechanisms and global structural response, demonstrate that the seismic performance of the cooling tower is controlled primarily by local material behavior rather than global dynamic characteristics. Spatial variability of ground motion activates complex deformation modes, leading to pronounced tensile damage, plastic strain accumulation, and stiffness degradation in the lower shell region. The structural variant with thickened lower zone of the shell exhibits extensive material deterioration, including the formation of a continuous plastic zone and irreversible deformation associated with damage localization. In contrast, the ring-stiffened configuration effectively limits damage propagation, reduces plastic strain by up to 80%, and maintains predominantly elastic material response with significantly lower stiffness degradation. The bottom ring stiffener is shown to provide superior performance by mitigating damage evolution of the concrete structure under spatially non-uniform seismic loading. The study highlights the critical role of advanced constitutive material modeling in capturing the realistic seismic behavior of reinforced concrete shell structures and demonstrates that structural strengthening strategies should be evaluated based on their ability to control material degradation mechanisms.

## 1. Introduction

Natural draft cooling towers are among the most demanding shell structures due to their large height, slender geometry, and high sensitivity to dynamic loading. Their design becomes particularly critical in regions of elevated seismic hazard. From a constructional perspective, hyperboloidal cooling towers commonly incorporate variable shell thickness, showing a reduction of thickness towards the throat and local thickening at the upper and lower parts, as exemplified by large-scale structures such as the Niederaussem [1], the Panipat [2], or the Pengze cooling towers [3]. These structural solutions are consistent with design recommendations, which require smooth thickness transitions, typically implemented as gradual (linear) tapering in the shell profile [4,5].

Design practice in seismic regions is based on guidelines requiring earthquake loads to be considered together with other actions, with seismic excitation explicitly identified as a critical load case in cooling tower analysis [5]. Due to the high sensitivity of hyperboloidal shell structures to dynamic effects, seismic loading requires nonlinear analysis [6]. Numerical studies confirm that thin-walled reinforced concrete hyperboloidal cooling towers are highly susceptible to seismic excitation and require advanced modelling methods, such as time-history analysis, to accurately assess their dynamic response [7].

Recent nonlinear seismic studies show that realistic assessment requires advanced constitutive modelling of reinforced concrete, including damage and plasticity effects [8], such as cracking, crushing, and progressive stiffness degradation developing during earthquake loading. Therefore, constitutive models capable of capturing these effects are essential for reliable simulation of structural response. The smeared crack concrete model has been used to describe failure processes in hyperbolic cooling towers, allowing identification of cracking and reinforcement yielding, particularly in wind-related analyses [9]. More advanced seismic studies commonly employ the Concrete Damaged Plasticity (CDP) model, often combined with reinforcement degradation models to account for corrosion effects and enable nonlinear dynamic response and fragility assessment [10]. Stochastic damage models based on energy principles and fiber bundle theory have also been developed to capture randomness in material behaviour and simulate progressive collapse mechanisms under strong earthquake excitation [11]. Comparisons with elastic analyses confirm that nonlinear modelling is necessary, as elastic approaches cannot reproduce cracking, yielding, and post-yield behaviour observed in reinforced concrete cooling towers during earthquakes [12].

Additionally, cooling towers are classified as multiple-support structures, and their large spatial extent makes them particularly sensitive to spatially varying earthquake ground motion (SVEGM) [13,14]. In such systems, differential support displacements induced by wave passage effects and incoherence of seismic waves can significantly alter internal force distribution and deformation mechanisms compared to the commonly assumed uniform excitation [15]. As a result, the structural response may involve additional deformation components, including increased shell ovalization and activation of higher circumferential vibration modes. This phenomenon is well documented in the literature. For example, asynchronous seismic excitation may increase the dynamic response and damage of the cooling tower shell compared to uniform excitation, indicating that the latter may be non-conservative [16]. Similarly, incorporating wave propagation effects and realistic ground motion records can amplify the dynamic response depending on soil conditions and wave characteristics [17].

Given these considerations, several important factors must be accounted for in the seismic analysis of cooling towers. However, the primary requirement remains the development of structural solutions that are inherently well adapted to seismic conditions. In contrast to standard design practice, cooling towers in seismic regions often require additional strengthening beyond conventional thickness variation. A simple linear thickness distribution may be insufficient; therefore, alternatives such as nonlinear (hyperbolic) thickness variation or a bottom ring stiffener are used. Both strengthening approaches are generally considered comparable in terms of material consumption and construction effort [18], so the selection depends primarily on structural performance under dynamic loading, i.e., which solution provides more effective seismic mitigation. For instance, in terms of stability, studies show that stiffening rings play an important role in controlling structural behaviour [19] and can significantly improve resistance to buckling [20].

The main objective of this study is to investigate the seismic response of a reinforced concrete cooling tower shell, with particular emphasis on the material modelling and its nonlinear constitutive behaviour, damage evolution, and stiffness degradation under spatially varying earthquake excitation. The study focuses on a comparative assessment of two strengthening strategies applied to the lower shell region–a locally thickened shell and a bottom ring stiffener–evaluated in terms of their effectiveness in controlling material deterioration mechanisms. The analysis is performed using nonlinear time-history simulations incorporating spatially varying earthquake ground motion (SVEGM) and a Concrete Damaged Plasticity (CDP) constitutive model. Special attention is given to the interaction between local material degradation processes (cracking, crushing, and plastic strain accumulation) and the global structural response. The key response measures include tensile damage, plastic strain evolution, stiffness degradation, and the development of localized failure mechanisms in the shell.

The novelty of this study lies in a material-oriented assessment of seismic performance of two alternative strengthening strategies for the lower part of a cooling tower–a locally thickened shell and a circumferential bottom ring stiffener–carried out under realistic seismic conditions. The proposed approach integrates: (i) advanced constitutive modelling of concrete using the CDP framework, enabling realistic representation of cracking and crushing mechanisms; (ii) spatially varying earthquake excitation, incorporating both wave passage and incoherence effects, calibrated using in-situ measurements; and (iii) nonlinear time-history analysis based on a real seismic record. This combination allows for a physically consistent evaluation of the coupling between spatially non-uniform loading and material degradation processes.

In contrast to existing studies, where strengthening strategies are assessed mainly in terms of global structural response, the present work evaluates their effectiveness based on their ability to control damage evolution and limit irreversible material degradation. This perspective provides new insight into the role of local stiffness distribution in controlling damage localization.

## 2. Materials and Methods

### 2.1. Multi-Support Structures Under Spatially Varying Earthquake Ground Motion

SVEGM arises from several physical mechanisms including: (i) the wave passage effect, resulting from the finite velocity of seismic waves and causing time delays between different locations; (ii) incoherence, which reflects the loss of coherence between ground motions due to scattering, reflections, and refractions in a heterogeneous medium; and (iii) local site effects, associated with variations in soil properties [13,21]. These phenomena become particularly significant in the analysis of spatially extended structures, where differential support excitation can strongly influence structural response. Such structures, characterized by large spatial extent in plan, are referred to as multiple-support structures.

The dynamic behavior of multi-degree-of-freedom systems subjected to seismic excitation is described by the coupled equations of motion that account for both structural and ground degrees of freedom [22,23]:

(1) $$\left[\begin{array}{cc} M_{ss} & M_{sg}\\ M_{gs} & M_{gg} \end{array}\right]\left\{\begin{array}{c} {\ddot{x}}_{s}\\ {\ddot{x}}_{g} \end{array}\right\}+\left[\begin{array}{cc} C_{ss} & C_{sg}\\ C_{gs} & C_{gg} \end{array}\right]\left\{\begin{array}{c} {\dot{x}}_{s}\\ {\dot{x}}_{g} \end{array}\right\}+\left[\begin{array}{cc} K_{ss} & K_{sg}\\ K_{gs} & K_{gg} \end{array}\right]\left\{\begin{array}{c} x_{s}\\ x_{g} \end{array}\right\}=\left\{\begin{array}{c} 0\\ F_{g} \end{array}\right\}$$

where:

s, g—degrees of freedom of structure and ground respectively,
M, C, K—mass, damping and stiffness matrices,
${\ddot{x}}_{s}$, ${\dot{x}}_{s}$, $x_{s}$—accelerations, velocities and displacements for each DOF of the structure,
${\ddot{x}}_{g}$, ${\dot{x}}_{g}$, $x_{g}$—accelerations, velocities and displacements for each DOF of the ground,
$F_{g}$—reaction vector.

The total nodal displacement vector can be decomposed into dynamic and quasi-static components [23]:

(2) $$x=\left\{\begin{array}{c} x_{s}^{d}\\ 0 \end{array}\right\}+\left\{\begin{array}{c} x_{s}^{q}\\ x_{g} \end{array}\right\}$$

where $x_{s}^{d}$ is the dynamic structural response and $x_{s}^{q}$ represents the quasi-static contribution induced by support displacements.

In the case of static structural behavior, the mass and damping matrices in Equation (1) and the dynamic component of motion in expression (2) vanish, which leads to the quasi-static displacements determination [22]:

(3) $$x_{s}^{q}=-K_{ss}^{-1}K_{sg}x_{g}$$

Substituting Equations (2) and (3) into Equation (1), the governing equation for the dynamic component becomes:

(4) $$M_{ss}{\ddot{x}}_{s}^{d}+C_{ss}{\dot{x}}_{s}^{d}+K_{ss}x_{s}^{d}=(M_{ss}K_{ss}^{-1}K_{sg}-M_{sg}){\ddot{x}}_{g}-(C_{ss}K_{ss}^{-1}K_{sg}-C_{sg}){\dot{x}}_{g}$$

According to Eurocode 8 [24], the velocity-dependent term can be neglected:

(5) $$M_{ss}{\ddot{x}}_{s}^{d}+C_{ss}{\dot{x}}_{s}^{d}+K_{ss}x_{s}^{d}=-\left(M_{ss}K_{ss}^{-1}K_{sg}+M_{sg}\right){\ddot{x}}_{g}$$

Equation (5) shows that the structural response is driven by spatially varying ground accelerations at the supports. Therefore, an accurate representation of support motion is essential in the analysis of multiple-support structures. Since seismic records are typically available at a single location, it is necessary to employ models that reproduce spatial variability.

The simplest approach is the delayed excitation (wave passage) model, which introduces time delays between supports based on the finite wave propagation velocity [23]. Although this model captures phase differences, it neglects incoherence effects associated with the loss of correlation between ground motions at different locations. Neglecting these effects may lead to unconservative estimates of structural response in multiple-support systems [13].

In this study, an advanced spatiotemporal seismic excitation model is adopted, incorporating both wave passage and incoherence effects, and providing a more realistic and reliable assessment of the dynamic response of multiple-support structures.

### 2.2. Conditional Random Field Simulation of Ground Motions for Multiple-Support Structures

This section introduces a methodology for conditional simulation of ground motion at multiple support locations of a structure, based on the spatiotemporal correlation framework proposed in [25,26,27]. This approach provides an efficient way to generate spatially variable seismic excitations using recorded earthquake data as input. For computational efficiency, the spatial correlation function is evaluated using only the dominant frequency of the seismic event, which significantly reduces the complexity of the simulation procedure.

The method, described in [25], allows the generation of acceleration time histories at locations that are not directly instrumented, using recorded seismic signals, assumed wave propagation velocity, and a prescribed spatial correlation model. The correlation function, describing the statistical dependence between ground motion values at two spatial points (*i* and *j*) is defined as:

(6) $$K(D_{ij})=\sigma^{2}e^{\left(\frac{-\omega_{d}\cdot D_{ij}}{2\pi v\alpha }\right)}$$

where:

*D_ij_*—separation distance between two field points (*i*, *j*),
*ω_d_*—predominant frequency of the shock,
*v*—wave velocity,
α—space scale parameter (*α* > 0), which controls the coherence drop and depends on local geological conditions,
σ—standard deviation of the recorded shock.

For two points, this correlation can also be expressed in matrix form:

(7) $$K=\left[\begin{array}{cc} K_{11} & K_{12}\\ K_{21} & K_{22} \end{array}\right]$$

To obtain a synthetic acceleration-time history (x_u_) at point *j*, on the basis of the known record (x_k_) at point *i*, the following sampling procedure is used:

(8) $$x_{u_{s}}=-a_{max}+2a_{max}\cdot r_{d},\;\;\;\;s=1,\cdots ,n$$

where:

$a_{max}$—maximum ground acceleration of the original record,
$r_{d}$—uniform random variable from a range <0, 1>.

Assuming a zero-mean Gaussian distribution of ground motion, the conditional probability density function for the truncated Gaussian field is given by:

(9) $$f(x_{u}|x_{k})=(1-l{)}^{-1/2}\;(detK_{c}{)}^{-1/2}\;(2\pi {)}^{-1/2}exp\left[-\frac{1}{2}(1-l)(x_{u}-m_{c}{)}^{T}K_{c}^{-1}\left(x_{u}-m_{c}\right)\right]$$

where:

$x_{u}$—vector of unknown values (generated signal),
$x_{k}$—vector of known values (registered signal),
$K_{c}=K_{11}-{K_{12}K}_{22}^{-1}\cdot K_{21}$—conditional covariance matrix of the random field,
$m_{c}={K_{12}K}_{22}^{-1}x_{k}$—vector of conditional mean values.
l—truncation parameter, dependent on the $\frac{a_{max}}{\sigma }$ ratio. When this ratio is 4.0 or greater, the truncation can be disregarded (l=0).

The created vector x_u_ is accepted only if it satisfies the von Neumann rejection condition:

(10) $$R\leq f(x_{u}|x_{k})$$

where:

(11) $$R=(detK_{c}{)}^{-1/2}(2\pi {)}^{-1/2}r_{d}$$

If the acceptance criterion is not met, a new vector x_u_ is generated and the procedure is repeated iteratively using the same formulation.

The application of this method requires two site-specific parameters: seismic wave velocity *v* and the spatial scale parameter α. While *v* describes wave propagation in the medium, α governs the spatial drop of correlation. Studies [25,26,27] show that α can vary considerably (0–100), depending on ground heterogeneity. High values indicate strong spatial coherence, whereas low values correspond to fractured or highly heterogeneous soils. In practice, α should be calibrated empirically from recorded seismic data.

### 2.3. Carpathian Flysch Seismicity and a Natural Seismic Shock Used for Dynamic Analyses

The Carpathian Flysch is a tectonically complex zone exhibiting moderate seismicity. This region hosts critical infrastructure, including power plants with cooling towers and other strategic facilities, which must be designed for seismic loading under moderate hazard conditions [24].

**Figure 1:**
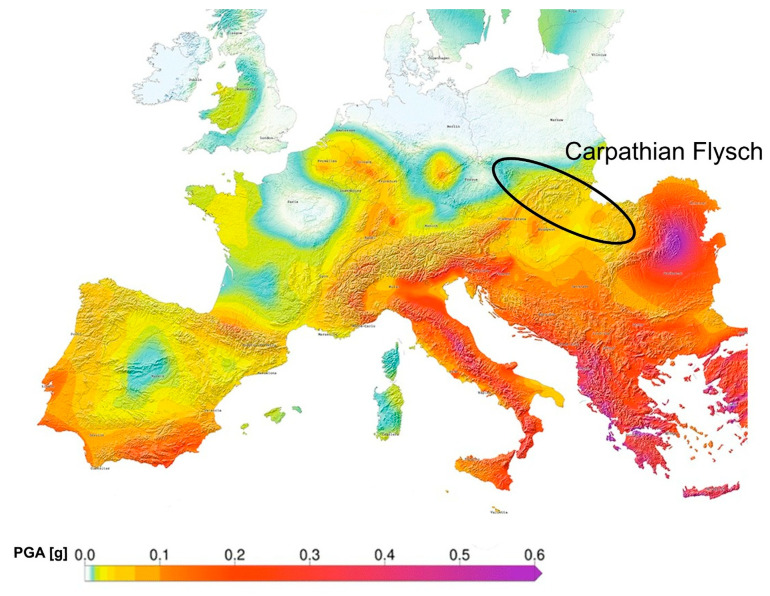
Seismic hazard map for Europe, including the Carpathian Flysch region, defined in terms of peak ground acceleration (PGA) and calculated with a 90% probability that acceleration values will not be exceeded over a 105-year period (1000-year return period) [28].

Figure 1 presents the seismic hazard map for Central and Northern Europe, including the Carpathian Flysch region [28]. It can be observed that Peak Ground Acceleration (PGA) in the region typically reaches 1.0–1.6 m/s^2^, reflecting higher expected ground motion compared to adjacent foreland areas.

Since 1997, ongoing observations have been carried out to assess local seismicity associated with the Carpathian Flysch near the Niedzica Dam, Southern Poland [29,30]. The monitoring system consists of three stations: Station A located approximately 1700 m west of the dam, and Stations B and C, installed in the dam’s inspection gallery within the bedrock. All stations are equipped with SM-3 seismometers and operate with 1 ms temporal resolution, enabling detailed analysis of seismic signals, including apparent wave velocity and loss of coherence [29,31,32]. The presented monitoring system registered a series of seismic events that occurred in November–December 2004 in the Carpathian Flysch region. The sequence exhibited swarm-type behaviour typical of the Western Carpathians, with multiple aftershocks occurring over a short time interval [33]. The most relevant event for dynamic analysis occurred on 2 December 2004, with a magnitude of 3.6. The epicenter of the shock was located in Czarny Dunajec, 33 km to the West of the stations.

**Figure 2:**
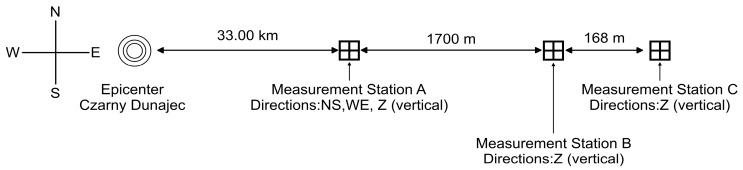
Layout of the seismic monitoring stations in the Carpathian Flysch region.

The layout of the seismic monitoring stations in the Carpathian Flysch with the location of epicenter of the analyzed seismic event are presented in Figure 2.

**Figure 3:**
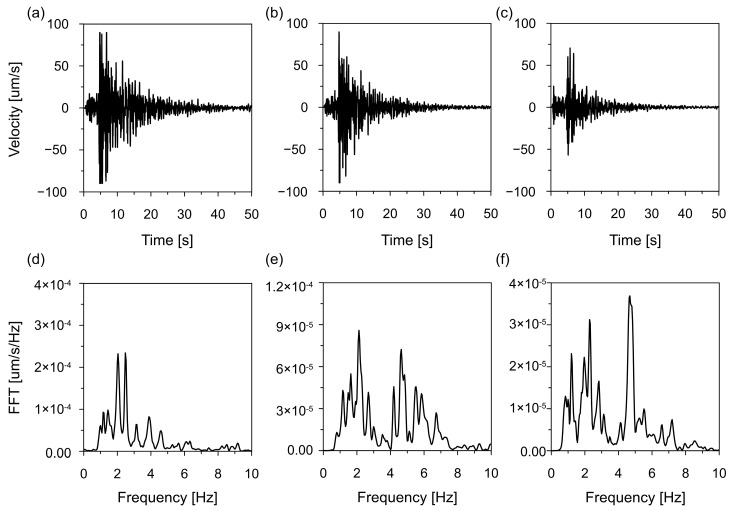
Ground vibration velocity and corresponding Fourier spectra of the seismic shock on 2 December 2004, recorded at Station A: (**a**) North–South (NS) direction, (**b**) West–East (WE) direction, (**c**) vertical (Z) direction; (**d**–**f**) Fourier spectra for the respective NS, WE, and Z components.

Figure 3 shows three-component earthquake recordings at Station A along with their corresponding Fourier spectra. The ground motion lasted approximately 30 s, including a strong shaking phase of about 15 s. Peak ground velocities reached around 90 mm/s in the horizontal direction and 70 mm/s in the vertical direction. The spectral content is dominated by frequencies between 1 and 3 Hz, with a primary peak at 2.3 Hz and a second peak in the 4–5 Hz range, which is consistent with regional seismic characteristics.

This earthquake record was used as kinematic input for the dynamic analyses of the cooling tower. The original velocity time history was first differentiated numerically to obtain the corresponding acceleration record and determine its PGA. Subsequently, a scaling factor, defined as the ratio of the target PGA value (1.6 m/s^2^) to the original PGA, was applied to the velocity time histories, preserving the original time-frequency characteristics of the signal while adjusting its intensity to the upper-bound seismic hazard level for the Carpathian region derived from seismic hazard assessments [28,34]. The event is considered representative due to its frequency content and is therefore suitable for evaluating the seismic response of strategic surface infrastructure under natural ground motion conditions in the Carpathian Flysch region [35,36,37].

### 2.4. Constitutive Parameters of the Concrete Damaged Plasticity Model

A critical aspect of dynamic structural analyses is the adoption of a suitable constitutive material model that accurately represents material behavior under seismic loading. To account for the nonlinear seismic response of the cooling tower, the CDP model was adopted.

The model is widely used in numerical simulations of concrete structures under cyclic and dynamic loading, as it effectively represents both irreversible plastic deformation and progressive stiffness degradation [38,39].

Within the CDP framework, the failure process is primarily governed by two dominant mechanisms: tensile cracking and compressive crushing. These two mechanisms are described through independent hardening/softening variables associated with tension and compression behaviour. The evolution of these variables defines the development of damage and the corresponding expansion of the yield surface during loading.

A key feature of the model is the degradation of elastic stiffness, which is represented by a scalar damage variable *d*. The effective elastic modulus is reduced according to the relationship:

(12) $$E=(1-d)E_{0}$$

where *E*_0_ denotes the initial (undamaged) Young’s modulus.

The damage parameter *d* is a combined measure of material deterioration resulting from both tensile and compressive damage mechanisms. In particular, the model distinguishes between tensile damage *d_t_* and compressive damage *d_c_*, each varying between 0 (undamaged state) and 1 (fully damaged state). The combined damage effect is commonly expressed as:

(13) $$\left(1-d)=(1-d_{c})(1-d_{t}\right)$$

The constitutive stress–strain relationship in the CDP model is defined in the effective stress space as:

(14) $$\sigma =(1-d)\;D_{0}^{el}:(\varepsilon -\varepsilon^{pl})$$

where σ is the stress tensor, $D_{0}^{el}$ is the initial elastic stiffness tensor, ε is the total strain tensor, and ε^pl^ represents the plastic strain component.

An important aspect of concrete behaviour under cyclic loading is stiffness recovery during load reversals. Experimental studies on concrete elements have shown that partial recovery of compressive stiffness occurs when cracks close during the transition from tension to compression. In contrast, tensile stiffness is not recovered when the loading path reverses. The Abaqus (Abaqus CAE 2022) [40] implementation of the CDP model allows the user to control stiffness recovery effects, enabling a more realistic representation of crack opening and closure under cyclic excitation.

In the present study, CDP material parameters were adopted from available experimental and literature-based calibrations [41]. The following basic mechanical properties of concrete were assumed: Young’s modulus *E* = 19.7 GPa, Poisson’s ratio *v* = 0.19, and mass density ρ = 2600 kg/m^3^. Additionally, the following values characterizing CDP in the Abaqus software were introduced: dilation angle $\psi =38^{\circ }$, eccentricity ϵ=0.1, $f_{b0}/f_{c0}=1.12$, $K_{c}=0.667$, and viscosity parameter μ=0.0. The remaining constitutive parameters describing concrete cracking, crushing, and stiffness degradation are listed in Table 1, which summarizes the compression hardening, tension stiffening, and damage evolution relationships adopted in the CDP model. The adopted parameter set represents experimentally calibrated nonlinear stress–strain behaviour of concrete under both compression and tension. In particular, compression hardening describes the evolution of compressive yielding and inelastic strains, while tension stiffening and damage parameters capture tensile cracking, stiffness degradation, and progressive loss of tensile load-carrying capacity under cyclic loading. These constitutive relationships are consistent with experimentally observed behaviour of reinforced concrete under seismic loading.

### 2.5. Structural Layout and Numerical Model Assembly of the Cooling Tower

Two structural variants of a natural draft cooling tower located in the Carpathian Flysch were considered in the numerical study:

(i) Variant 1—a tower with a locally thickened shell in the lower region, and
(ii) Variant 2—a tower equipped with a bottom ring stiffener.

**Figure 4:**
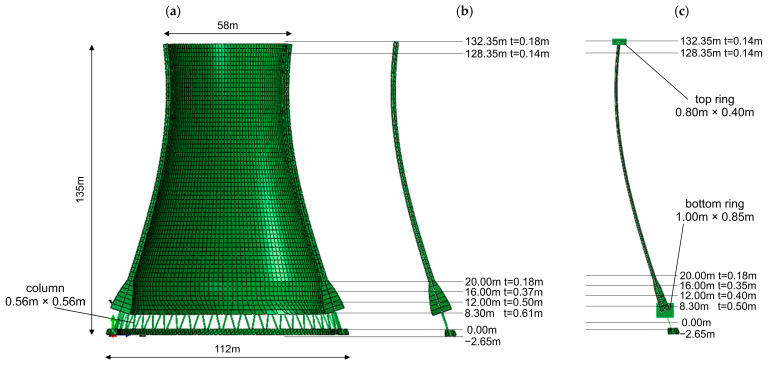
(**a**) The numerical model of the cooling tower along with main dimensions, (**b**) structural solution with locally thickened shell in the lower region, (**c**) structural solution with a bottom ring stiffener.

Both models share the same global geometry, including the foundation system and supporting columns, and differ only in the structural solution adopted in the lower shell zone. The height of the tower is 135 m, the bottom diameter is 112 m, the middle diameter is 53 m, and the top diameter is 58 m. The structure is supported by reinforced concrete columns with a cross-section of 56 cm × 56 cm, while the foundation block has a height of 2.65 m and a width of 5 m. The numerical model of the cooling tower, including its main dimensions and both structural variants, is presented in Figure 4.

The thickness of the shell depends on the variant of the analyzed structure. In the case of the thickened-shell variant, the shell thickness in the upper part varies linearly from 18 cm at a height of 132.35 m to 14 cm at 128.35 m. From this level down to 20 m, the thickness remains constant at 18 cm. In the lower region, the thickness increases hyperbolically from 18 cm at a height of 20 m to 61 cm at a height of 8.3 m.

For the ring-stiffened variant, the shell thickness in the upper part is constant at 14 cm. A ring beam with dimensions of 0.8 m × 0.4 m is located at the top edge of the structure. From this level down to 20 m, the shell thickness is the same as in Variant 1 (18 cm). In the lower region, the variation in thickness is less pronounced, increasing linearly from 18 cm to 50 cm. Additionally, a circumferential ring beam with a cross-section of 1.0 m × 0.85 m is introduced at the base of the shell.

The total structural mass of the cooling tower without the ring is 26,101 tons, whereas the mass of the structure with the bottom ring is 26,213 tons. The difference is therefore only 112 tons, corresponding to approximately 0.4% of the total mass. Such a small discrepancy indicates that the concrete consumption in both variants is practically identical, and consequently, the two solutions can be regarded as economically equivalent in terms of material costs.

Two variants of numerical model were developed using the finite element method in Abaqus [40]. Different element types were used to represent various parts of the structure. The cooling tower shell was modeled using four-node reduced-integration shell elements (S4R), the supporting columns and ring stiffeners were modeled using two-node beam elements (B31), and the foundation block was discretized using four-node tetrahedral solid elements (C3D4). In the case of the model with a locally thickened shell, the total number of elements was 12,111. For the model with the bottom ring, the total number of elements increased slightly to 12,355 due to the inclusion of additional elements representing the ring. The structure was assumed to be fixed at the base, and soil–structure interaction was not considered in this stage of the analysis.

The material behavior of concrete was described using the CDP model, which allows for realistic simulation of nonlinear behavior, including cracking in tension and crushing in compression. The adopted parameters are presented in Table 1 (see Section 2.4). Reinforcement was not explicitly modelled as discrete rebar elements in the finite element formulation. Instead, its effect was incorporated through a homogenized concrete–steel material response using an effective Young’s modulus approach, assuming a reinforcement ratio of approximately 3%, which resulted in an equivalent Young’s modulus of $E_{\mathrm{eff}}\approx 25.0\;\mathrm{GPa}$.

### 2.6. Limitations of the Study

Despite the comprehensive nonlinear numerical framework adopted in this study, several limitations should be acknowledged when interpreting the obtained results.

First, the analysis assumes a fully fixed-base condition and therefore neglects soil–structure interaction (SSI). While this simplification reduces model complexity and enables a clearer comparison between the two structural variants, it may affect the absolute response levels by neglecting foundation flexibility, soil damping, and possible changes in the natural vibration characteristics. However, considering the relatively stiff bedrock conditions of the Carpathian Flysch formation, this assumption is regarded as acceptable for the present comparative study.

Second, the seismic assessment is based on a single real earthquake record from the Carpathian Flysch region, scaled to the target peak ground acceleration representative of regional seismic hazard conditions. Although the selected event is well documented and representative in terms of frequency content and regional seismicity, the use of a single ground motion limits the statistical generalizability of the results. In accordance with seismic design practice (e.g., Eurocode 8 and ASCE 7), multiple records are generally required to obtain statistically reliable response measures. In the present study, however, the use of a single record was intentional in order to isolate the influence of structural configuration and material nonlinearity without introducing additional stochastic variability.

Third, the spatially varying seismic excitation model relies on calibrated parameters such as wave velocity and spatial correlation scale derived from available field measurements. Although these parameters are site-specific, their estimation remains subject to uncertainty, which may influence the exact spatial distribution of support excitation.

Finally, the adopted CDP parameters were taken from established literature sources and applied consistently to both structural variants, but the model was not directly validated against full-scale experimental data. Consequently, the study should be interpreted primarily as a comparative and mechanistic investigation focused on relative differences in structural response, damage evolution, and stiffness degradation, rather than as a fully predictive assessment of real structural behavior.

Future studies incorporating coupled SSI effects, multiple earthquake scenarios, and experimental validation would further improve the applicability of the proposed seismic assessment framework.

## 3. Results and Discussion

### 3.1. Determination of Wave Velocity and Space Scale Parameter for Carpathian Flysch Bedrock Conditions

The initial stage of the analysis focused on determining the seismic wave propagation velocity and the space scale parameter for the Carpathian Flysch formation based on real earthquake recordings.

The apparent wave velocity *v* can be estimated using the cross-correlation function of two signals *x*(*t*) and *y*(*t*) measured along the wave path:

(15) $$K(\tau )=\underset{\tau }{max}\int_{-T/2}^{T/2}x(t)\;y(t+\tau )\;dt$$

The peak of K(τ) occurs at the time delay τ, representing the travel time between sensors. Knowing the distance *d*, the apparent wave velocity is:

(16) $$v=\frac{d}{\tau }$$

The delay τ corresponds to the best temporal alignment of *x*(*t*) and y(t+τ). The resulting velocity represents an average velocity of a multi-frequency wave signal.

**Figure 5:**
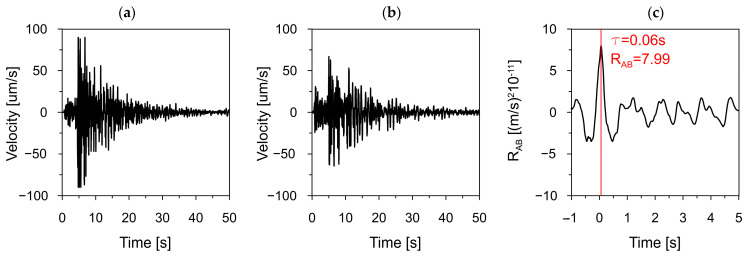
Time-histories of velocities in vertical direction (Z) recorded at: (**a**) Station B, (**b**) Station C, and their cross-correlation function (**c**).

To evaluate wave propagation velocity for the Carpathian Flysch two measurement points were considered–Station B and Station C (see Section 2.3, Figure 2). The time-histories of velocities recorded at these stations and the cross-correlation function between them are presented in Figure 5.

The cross-correlation function showed a clear maximum at 0.06 s, corresponding to the time delay associated with seismic wave propagation between the sensors. This delay represents the travel time of the seismic wave through the bedrock. Based on this time delay (0.06 s) and the known distance between the sensors (168 m), the apparent seismic wave velocity was determined as *v* = 2800 m/s. This value is considered typical for rock masses occurring within the Carpathian Flysch, composed predominantly of marls, limestones, and radiolarites [42,43,44].

Using the peak value of the cross-correlation function, the spatial scale parameter α was determined based on the adopted analytical formulation (see Section 2.2, Equation (6)). For the flysch bedrock conditions, the following parameters were used:

- distance between sensors: D=168 m,
- dominant frequency of the seismic excitation: $\omega_{d}=2.31\;\mathrm{Hz}$,
- seismic wave velocity: v=2800 m/s,
- standard deviation of the signal at Station A: $\sigma =1.01\times 10^{-5}$,
- maximum cross-correlation value: K(168 m)=7.99.

Based on these data, the estimated value of the space scale parameter is α = 0.43. This parameter characterizes the rate at which the spatial correlation of seismic motion drops with distance. The obtained value indicates a moderate loss of coherence over increasing separation between measurement points. Such behavior is consistent with the heterogeneous nature of the Carpathian Flysch rock mass, where structural complexity enhances the scattering and attenuation of seismic waves, resulting in reduced spatial correlation between recordings. Top of Form, Bottom of Form.

### 3.2. Dynamic Characteristics of the Cooling Tower

Before performing the seismic response analysis of both variants, a modal analysis was carried out to determine the natural frequencies and corresponding mode shapes. The results indicate that the dynamic properties of the two configurations are very similar.

The vibration modes of cooling towers exhibit characteristic deformation patterns typical for thin-walled shell structures. The lowest modes are associated with circumferential deformation, in which the shell undergoes ovalization and forms wave-like patterns along its perimeter. These mode shapes can be described using the circumferential wave number *n*, which defines the number of deformation waves distributed along the shell circumference. For *n* = 1, no ovalization occurs and the response corresponds to a rigid-body-type deformation, whereas ovalization appears for *n* ≥ 2. In particular, *n* = 2 represents an elliptical deformation shape, while higher values (e.g., *n* = 3, 4) lead to multi-lobed patterns.

**Figure 6:**
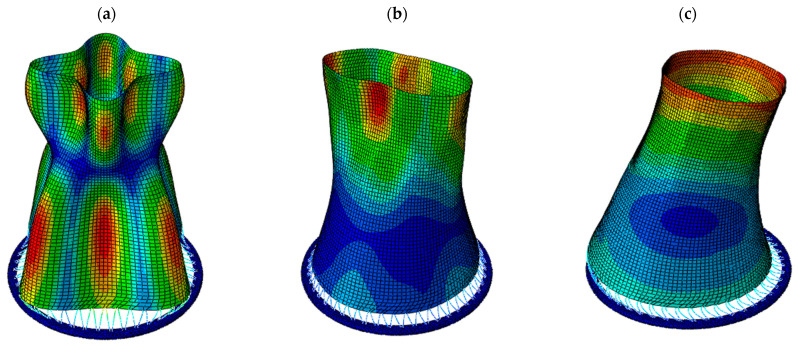
Natural vibration modes of the tower: (**a**) the lowest mode at frequency 0.86 Hz, associated with a 4th harmonic; (**b**) the mode at frequency 1.58 Hz, associated with shell ovalization; (**c**) the mode at frequency 3.07 Hz, associated with global bending mode (the displacement values are marked with colors: from blue for zero displacement to red for the absolute maximum displacement).

Representative vibration modes of the cooling tower for both structural variants are shown in Figure 6. For the tower with the thickened lower shell, the first natural frequency is 0.86 Hz and corresponds to a circumferential mode associated with a high-order harmonic (see Figure 6a). The ovalization mode with *n* = 2, corresponding to an elliptical deformation shape, occurs at a frequency of 1.58 Hz (see Figure 6b). The global bending mode of the structure (see Figure 6c) is associated with a frequency of 3.07 Hz. For the tower with the bottom ring stiffener, the corresponding frequencies are 1.57 Hz, 0.81 Hz, and 3.02 Hz, respectively.

The modal analysis proved that the introduction of the bottom ring has only a minor influence on the natural frequencies and mode shapes. Therefore, the differences observed in the seismic response of the two variants can be attributed primarily to variations in stiffness distribution and nonlinear behavior in the lower shell region, rather than to changes in global dynamic properties.

### 3.3. Uniform vs. Non-Uniform Kinematic Excitation: Effects on Cooling Tower Ovalization

**Figure 7:**
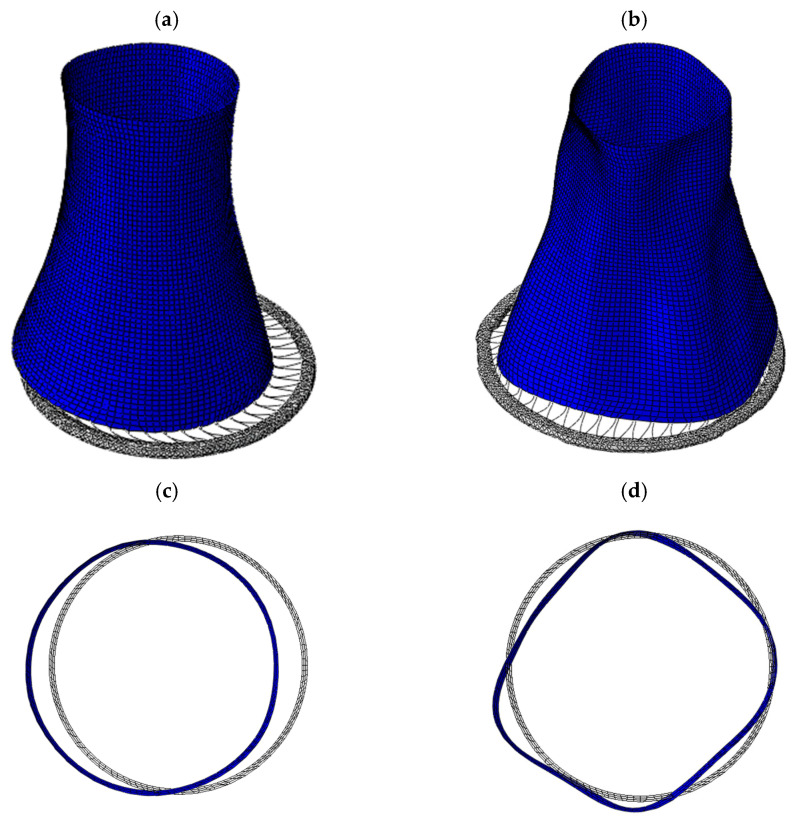
The deformation pattern of the tower under (**a**) uniform and (**b**) non-uniform excitation, together with detailed views of the bottom ring deformation (**c**,**d**) at 4.72 s of the shock.

The global deformation patterns and the deformation of the bottom part of the shell for both variants of the cooling tower are shown in Figure 7.

Under uniform kinematic excitation, the deformation pattern of the whole cooling tower is dominated by the global bending mode (see Figure 7a). The circular geometry of the whole shell is largely preserved, and the cross-section remains nearly circular, indicating that the response is primarily governed by the non-ovalization effects (*n* = 1). The contribution of the ovalization mode (*n* = 2) of the base is minimal (see Figure 7c), and higher modes (*n* ≥ 3) are not activated. This confirms that uniform excitation does not introduce substantial circumferential distortions in the structure.

In contrast, under SVEGM, the deformation pattern of the whole structure exhibits clear participation of higher circumferential modes (see Figure 7b). The cross-section undergoes ovalization, and the distortions observed in the bottom part of the shell (see Figure 7d) indicate activation of higher-order modes (*n* ≥ 2). Moreover, the deformation becomes strongly asymmetric, with certain regions experiencing significantly larger radial displacements than others.

**Figure 8:**
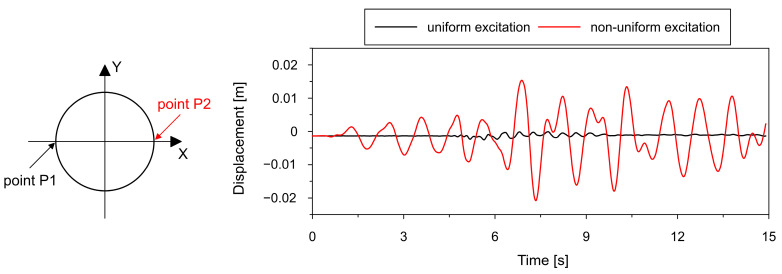
A comparison of changes in the chimney bottom diameter along the X-axis for both the uniform and non-uniform excitation models.

This observation is further confirmed by the results presented in Figure 8, which shows the variation of the chimney bottom diameter over time along the X-axis. For uniform excitation (black line), the changes in diameter are relatively small and symmetric, indicating negligible ovalization and preservation of the circular cross-section. In contrast, for SVEGM (red line), the variation in bottom diameter is considerably larger and asymmetric, clearly demonstrating the development of ovalization effects.

### 3.4. Comparative Assessment of Thickened Lower Shell and Bottom Ring Stiffener Considering Nonlinear Material Behavior in Cooling Tower Dynamics Under SVEGM

The dynamic responses of the cooling tower to the seismic shock were calculated using the Time History Analysis (THA) with the Hilber–Hughes–Taylor time integration algorithm, employed in the Abaqus software for a direct step-by-step solution. The THA simulations incorporated Rayleigh damping, based on mass and stiffness proportionality [45]. Assuming the critical damping ratio of 5% at the lowest frequency of 0.86 Hz and at the frequency of 3.07 Hz, accompanied by the first global bending, the coefficients of the Rayleigh damping model were estimated as 0.41 for the mass and 0.004 for the stiffness proportional damping. The dynamic response level was assessed using the tensile equivalent plastic strain (PEEQT), tensile damage parameter (DAMAGET), and logarithmic maximum principal strain (LEPmax). The logarithmic strain measure was adopted to ensure an accurate representation of large deformations and strongly nonlinear material behavior, while the equivalent plastic tensile strain (PEEQT) was used to describe the accumulation of plastic deformation.

The results presented in Figure 9, Figure 10, Figure 11, Figure 12, Figure 13, Figure 14, Figure 15, Figure 16 and Figure 17 provide a comprehensive comparison of the dynamic response of two cooling tower structural variants subjected to SVEGM: a tower with a thickened lower shell and a tower equipped with a bottom ring stiffener.

**Figure 9:**
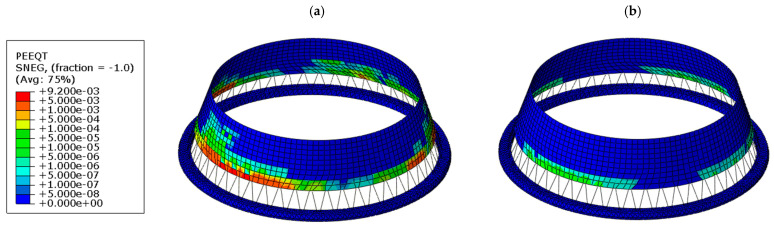
Comparison of tensile equivalent plastic strain (PEEQT) distribution in the lower shell region for two cooling tower variants: (**a**) thickened lower shell, (**b**) bottom ring stiffener.

**Figure 10:**
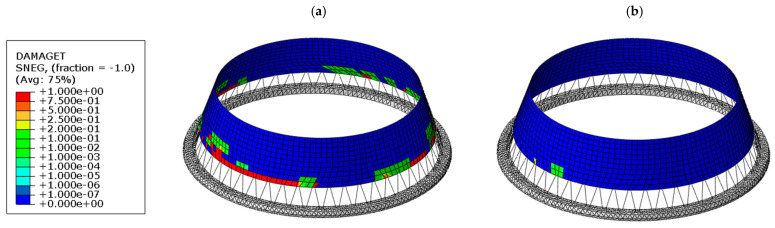
Comparison of tensile damage parameter (DAMAGET) distribution in the lower shell region for two cooling tower variants: (**a**) thickened lower shell, (**b**) bottom ring stiffener.

Figure 9 illustrates the distribution of tensile equivalent plastic strain (PEEQT) accumulated during the whole quake in the lower shell region. In the case of the thickened shell (see Figure 9a), a continuous circumferential zone of plastic strain develops near the base, indicating extensive yielding of the material. In contrast, the bottom ring configuration (see Figure 9b) significantly reduces both the magnitude and spatial extent of plastic strains, which remain localized in very limited area. This demonstrates that the ring effectively restrains the development of inelastic deformation. Quantitatively, the application of the bottom ring leads to an approximately 80% reduction in plastic strain compared to the thickened shell variant. A similar trend is observed in Figure 10, which presents the tensile damage parameter (DAMAGET). The thickened shell exhibits widespread damage concentrated in the lower region (see Figure 10a), whereas the ring-stiffened structure shows a clear reduction in both the intensity and extent of damage (see Figure 10b).

**Figure 11:**
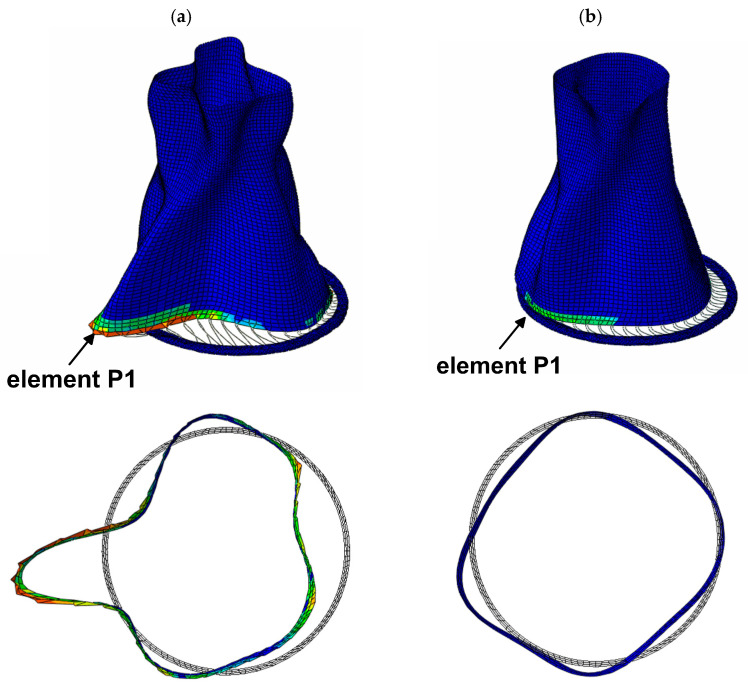
Comparison of global shell deformations at *t* = 6.58 s and accompanying lower part ovalization for two cooling tower variants: (**a**) thickened lower shell (with evident plastic zone), (**b**) bottom ring stiffener (the colours represent the tensile equivalent plastic strain PEEQT—see Figure 9).

Further insight into the structural behavior is provided by Figure 11, which shows the global deformation patterns as well as the deformation of the lower part of the tower at time *t* = 6.58 s, immediately after the strongest phase of the seismic excitation. In the thickened shell variant, a localized plastic zone develops in the lower part of the structure (element P1), accompanied by pronounced outward radial deformation, shell ovalization, and local shell bulging (see Figure 11a). This behavior is associated with the localization of plastic strains, stiffness degradation, and accompanying local shell deformation in the lower shell region. In contrast, the bottom ring prevents the formation of such a hinge, resulting in a smoother deformation profile and reduced ovalization of the shell (see Figure 11b). This confirms that the ring enhances circumferential stiffness and stabilizes the structural response more effectively than the thickened shell.

The comparison of the two cases demonstrates that spatially varying excitation leads to significantly more complex deformation patterns, including noticeable ovalization and multi-lobed shapes, which are not captured under the assumption of uniform excitation. Spatially varying excitation not only amplifies deformation but also fundamentally alters the modal composition of the seismic response by activating circumferential modes that are otherwise negligible under uniform loading conditions. These phenomena are particularly evident in the lower part of the shell, where boundary constraints amplify the influence of spatial variability in excitation.

**Figure 12:**
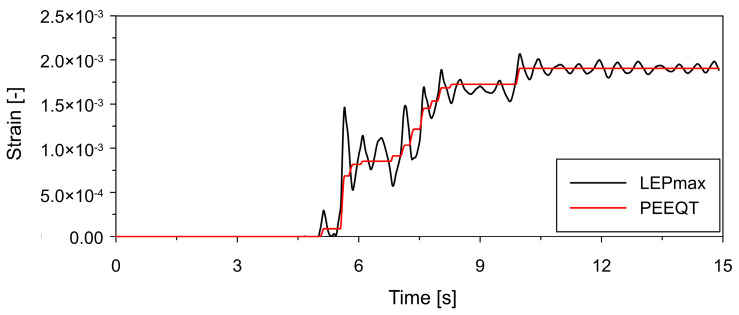
Time histories of tensile equivalent plastic strain (PEEQT) along with logarithmic maximal principal strain (LEPmax) at point P1 (see Figure 11) located in the lower shell region for thickened lower shell variant.

**Figure 13:**
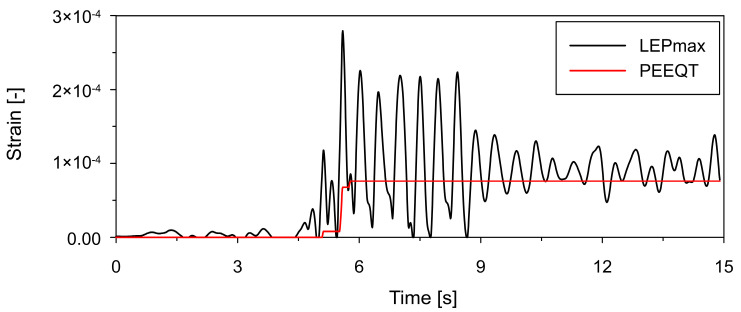
Time histories of tensile equivalent plastic strain (PEEQT) along with logarithmic maximal principal strain (LEPmax) at point P1 (see Figure 11) located in the lower shell region for bottom ring stiffener variant.

In both structural variants, plastic deformation develops in the lower part of the shell. Time-history responses at point P1, located in the most critical region of the lower shell, are shown in Figure 12 and Figure 13 for the thickened and ring-stiffened variants, respectively.

For the thickened shell (see Figure 12), PEEQT increases progressively over time, indicating continuous accumulation of plastic strain. In contrast, the ring-stiffened variant (see Figure 13) exhibits an evident step-like increase in PEEQT during the phase of high-amplitude excitation. In both cases, LEPmax follows a similar trend.

**Figure 14:**
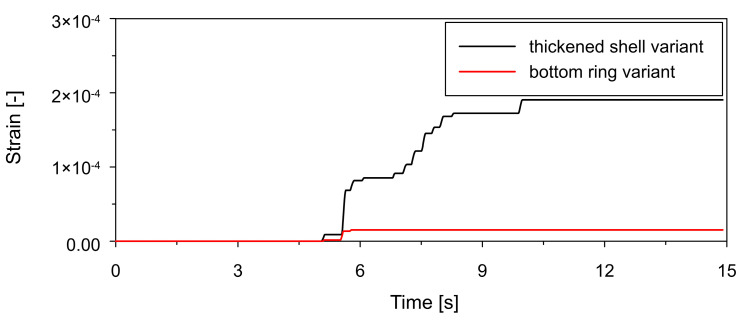
Comparison of time histories of tensile equivalent plastic strain (PEEQT) at point P1 (see Figure 11) located in the lower shell region for thickened lower shell variant (black line) and bottom ring stiffener variant (red line).

**Figure 15:**
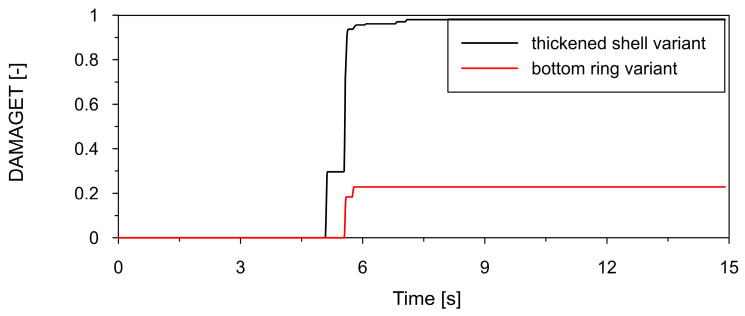
Comparison of time histories of tensile damage parameter (DAMAGET) at point P1 (see Figure 11) located in the lower shell region for thickened lower shell variant (black line) and bottom ring stiffener variant (red line).

A direct comparison of tensile equivalent plastic strain (PEEQT) is presented in Figure 14, where the thickened shell develops plastic strains approximately one order of magnitude higher than the ring-stiffened configuration. This indicates that the ring effectively reduces the maximum plastic strain, leading to improved control of inelastic deformation. The evolution of the tensile damage parameter (DAMAGET), presented in Figure 15, also confirms this trend: the thickened shell exhibits earlier damage initiation and higher damage levels, whereas the ring-stiffened delays damage onset and limits its magnitude.

**Figure 16:**
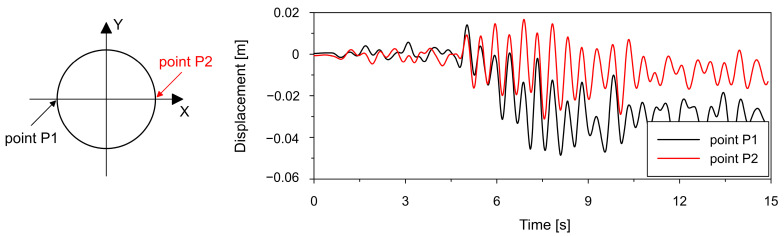
Time histories of displacements in horizontal direction X at points P1 and P2 located in the lower shell region for thickened lower shell variant.

**Figure 17:**
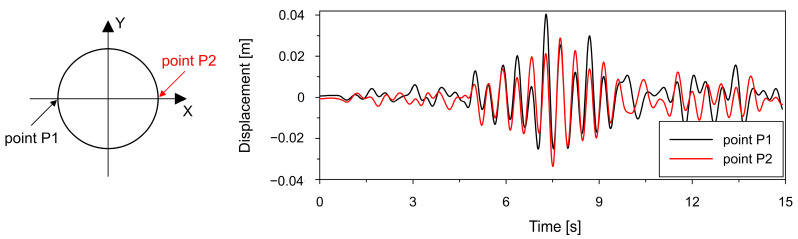
Time histories of displacements in horizontal direction X at points P1 and P2 located in the lower shell region for bottom ring stiffener variant.

Figure 16 and Figure 17, which present time histories of horizontal displacements (X direction) at selected points in the lower shell, clearly demonstrate a fundamental difference in the displacement response of the two cooling tower variants.

For the thickened lower shell (see Figure 16), the displacement histories in the X direction at points P1 and P2 reveal a permanent offset, especially at P1. After an initial oscillatory response around the original position, the structure shifts to a new equilibrium displaced by approximately 3 cm. This indicates plastic zones and irreversible deformations in the shell geometry.

In contrast, the bottom ring variant (see Figure 17) exhibits purely oscillatory behavior. The displacement time histories remain centered around the original position, with no residual displacements. This confirms that the bottom ring prevents plastic hinge formation and effectively limits permanent deformations, resulting in a predominantly elastic structural response.

Overall, the presented results demonstrate that the bottom ring stiffener significantly enhances the seismic performance of the cooling tower. Compared to the thickened lower shell, it reduces plastic deformations and damage, prevents the formation of plastic hinges, limits ovalization effects, and decreases displacement amplitudes. These improvements indicate a more favorable distribution of internal forces and a substantially lower risk of structural failure under seismic excitation.

## 4. Conclusions

This study presents a material-oriented interpretation of the seismic response of reinforced concrete cooling tower shells, considering two structural configurations: a locally thickened shell and a bottom ring-stiffened solution. The analysis accounts for spatial variability of earthquake excitation (SVEGM). Based on nonlinear dynamic simulations employing the Concrete Damaged Plasticity (CDP) model, the following conclusions can be drawn:

1. The application of the CDP constitutive model proves essential for capturing realistic seismic behavior, as linear or simplified approaches cannot reproduce the observed damage evolution and stiffness loss. Accurate representation of material nonlinearity is therefore critical for reliable assessment of reinforced concrete shell structures.
2. The seismic response of the cooling tower is governed primarily by local material behavior, rather than global dynamic characteristics. The results demonstrate that tensile damage evolution, plastic strain accumulation, and stiffness degradation control the overall structural performance. Spatially varying earthquake excitation significantly intensifies material degradation mechanisms. Wave passage and incoherence effects activate complex deformation modes, leading to increased cracking, damage localization, and non-uniform distribution of plastic strains, particularly in the lower shell region.
3. The locally thickened shell configuration does not effectively prevent damage accumulation, as it leads to the formation of extended plastic zones and irreversible material degradation. The increased thickness modifies stiffness but does not sufficiently limit tensile damage propagation.
4. The bottom ring-stiffened configuration provides a substantially improved material response, as it reduces tensile damage, limits plastic strain development, and mitigates stiffness degradation. The presence of the ring stiffener alters the stress distribution, preventing the formation of continuous damage zones and maintaining predominantly elastic behavior in critical regions.

Overall, the results demonstrate that the seismic response of the cooling tower under spatially varying excitation is primarily determined by nonlinear material behavior and damage evolution in the lower shell region, highlighting the decisive role of material modeling in controlling inelastic deformation and assessing the effectiveness of strengthening strategies.

## Figures and Tables

**Table 1:** Parameters describing the nonlinear behaviour of concrete in the CDP model [41].

**Concrete Compression** **Hardening**		**Concrete Compression** **Damage**	
**Compression** **Yield Stress [kPa]**	**Crushing Strain** **[-]**	**Compression Damage Parameter [-]**	**Crushing Strain** **[-]**
15,000	0.00	0.00	0.00
20,190	7.473 × 10^−5^	0.00	7.473 × 10^−5^
30,000	9.884 × 10^−5^	0.00	9.884 × 10^−5^
40,300	0.0001541	0.00	0.0001541
50,000	0.0007615	0.00	0.0007615
40,230	0.0025575	0.19	0.0025575
20,230	0.0056754	0.60	0.0056754
5250	0.0117331	0.89	0.0117331
**Concrete Tension** **Stiffening**		**Concrete Tension** **Damage**	
**Tension Yield Stress [kPa]**	**Cracking Strain** **[-]**	**Tension Damage Parameter [-]**	**Cracking Strain** **[-]**
1998.93	0.00	0.00	0.00
2842.00	3.333 × 10^−5^	0.00	3.333 × 10^−5^
1869.81	0.0001604	0.41	0.0001604
862.72	0.0002797	0.69	0.0002797
226.25	0.0006845	0.92	0.0006845
56.57	0.0010867	0.98	0.0010867

## Data Availability

The original contributions presented in this study are included in the article. Further inquiries can be directed to the corresponding author.
